# Cystic fibrosis patient characteristics and healthcare resource utilization in Finland using linked registries

**DOI:** 10.1016/j.heliyon.2024.e33439

**Published:** 2024-06-22

**Authors:** Kirsi Malmivaara, Mari Pölkki, Tuire Prami, Paavo Raittinen, Eija Heikkilä, Antti Aalto, Teija Dunder, Varpu Elenius, Kristina Sandström, Lisa J. McGarry

**Affiliations:** a Helsinki University Hospital, Helsinki, Finland; b Oriola, Espoo, Finland; c Swell Scientific, Helsinki, Finland; d Oulu University Hospital, Oulu, Finland; e Turku University Hospital, Turku, Finland; f Vertex Pharmaceuticals AB, Stockholm, Sweden; g Vertex Pharmaceuticals Incorporated, Boston, MA, USA

**Keywords:** Cystic fibrosis, healthcare resource use, Finland, Retrospective registry study, Matched cohort study

## Abstract

**Background:**

Knowledge of prevalence and epidemiology of cystic fibrosis (CF) and healthcare resource use among Finnish people with CF is incomplete.

**Methods:**

We conducted a population-based matched cohort study using retrospective real-world data from linked Finnish national registries. Electronic healthcare data and drug purchases of 102 people with CF were analyzed between January 2015 and December 2019 (follow-up). A 5-fold control population was matched by sex, age, and place of residence. Comorbidities and medication use that occurred at any time during follow-up were assessed; annual rates of hospital service use were adjusted for follow-up.

**Results:**

The prevalence of CF in Finland was 1.85 per 100,000. Median age at diagnosis was 1 year, with 60 % diagnosed at age <2 years and 80 % at age <10 years. Median age at death in people with CF was 31.4 years (n < 5); no controls died. The most common comorbidities included chronic sinusitis (39.2 %), pneumonia (38.2 %), diabetes (20.6 %), and nasal polyps (18.6 %). The most purchased medications were antibiotics (99.0 %) and pancreatic enzymes (84.3 %). The annualized rate of hospital visits was higher in people with CF vs controls (outpatient: mean [SD], 17.4 [14.5] vs 0.9 [3.3]; median, 13.6 vs 0.4, respectively; inpatient: mean [SD], 1.0 [1.66] vs 0.03 [0.14]; median, 0.34 vs 0, respectively).

**Conclusions:**

The prevalence of CF in Finland is remarkably low, likely reflecting unique population characteristics and, in part, delayed diagnosis. Antibiotic use is frequent among Finnish people with CF. Inpatient hospital visits are >30 times higher in people with CF than matched controls.

## Highlights

• The prevalence of diagnosed CF in Finland (1.85/100,000) was low vs other countries.
• Due to the lack of newborn screening, only 60 % of cases were diagnosed at age <2 years.
• The median age at death was lower in people with CF (31yrs) vs the Finnish population.
• Inpatient hospitalizations were >30 times more frequent in CF vs control population.
• In Finland, CF disease burden may improve with increased newborn screening and CFTRm therapy.

## 1 Introduction

Cystic fibrosis (CF) is an autosomal recessive disease that is caused by mutations in the CF transmembrane conductance regulator (*CFTR*) gene, which codes for a chloride channel [1,2]. Different types of mutations lead to various defects in the CFTR protein (eg, defective synthesis, processing, folding, trafficking, maturation, gating, or conductance and reduced abundance or stability) and can have distinct effects on disease severity [2].

CFTR defects affect the function of multiple vital organs, such as the pancreas and intestine, but particularly the lungs [3]. In the respiratory system, viscous mucus obstructs airways and makes them prone to infection and inflammation [4]. Additionally, CF-related diabetes is one of the most common CF-related comorbidities and is diagnosed in up to half of adults with CF [5].

CF is a progressive disease that eventually leads to premature death [4]. In recent decades, treatment has evolved and improved, initially mainly due to development of diagnostics and supportive therapies [4]. The most significant recent improvement is mutation-specific CFTR modulator (CFTRm) therapy, which addresses the molecular basis of CF [4]. In 2005, 14 CFTR mutations comprised 97 % of all mutations in Finland, of which *F508del-CFTR* was the most common (36 % of *CFTR* alleles) [6].

Finland has 5.5 million residents [7] and a healthcare system that is free to all Finnish citizens. New CFTRm therapies have been available with reimbursement in Finland since early 2021: 1) the combination of lumacaftor and ivacaftor (Orkambi®) for people aged ≥2 years with CF who are homozygous for *F508del-CFTR* (*F/F* genotype); 2) the combination of tezacaftor and ivacaftor (Symkevi®) for people aged ≥6 years with CF who have the *F/F* genotype or are heterozygous for *F508del-CFTR* and a residual function mutation; and 3) the triple combination of elexacaftor, tezacaftor, and ivacaftor (Kaftrio®) for people aged ≥6 years with CF with ≥1 copy of *F508del-CFTR* [[8], [9], [10], [11]]. Since *F508del-CFTR* is the most common mutation in Finland [6], many people with CF in Finland now have meaningful therapeutic options to treat the underlying cause of a hitherto lethal disease. However, knowledge of the prevalence and epidemiology of CF and healthcare resource use among Finnish people with CF is incomplete. Due to regional isolation and sparse inhabitation, the Finnish population has a unique disease heritage [6]. In previous studies, CF prevalence in Finland was estimated at 1:25,000 per newborn, which is one of the lowest in Caucasian populations [12].

Finland did not have a CF-specific patient registry when this study was conducted. However, in Finland, as in other Nordic countries, legislation allows the use of data from various national healthcare registries for research purposes [13]. These national registries are collected primarily for administrative purposes, but allow linkage of demographic, diagnostic and healthcare utilization data for scientific purposes. All citizens have a unique identification number that allows data collected from national registries to be linked at the individual level. The Finnish Social and Health Data Permit Authority (Findata) was established in 2020 to centralize and facilitate secure use of social and health care data for research [14]. Here, we describe CF epidemiology and healthcare resource use among people with CF in Finland by extracting and combining data from several Finnish national registries managed by Findata, along with CF-specific registry data from five university hospitals in Finland: Helsinki, Kuopio, Oulu, Tampere, and Turku. Each of these five university hospitals has established their own data lake that includes detailed patient data extracted from different electronic medical records. Our results describe the situation before CFTRm therapy was approved in Finland and will serve as an important benchmark for future studies.

## 2 Materials and methods

This was a population-based matched cohort study based on retrospective real-world data. The study cohort consisted of people with CF and matched individuals from the Finnish general non-CF population. Data for cohort members were assembled from several Finnish national registries combined with 5 university hospitals’ electronic records (data lakes; Table 1). The primary study objectives were to estimate the CF prevalence in Finland in 2019, characterize demographics and median age at death in people with CF, and document the impact of CF on comorbidities and healthcare resource use among people with CF compared with matched non-CF controls. Permission for data handling was provided by Findata (diary numbers: THL/6256/14.02.00/2020; THL/3657/14.06.00/2021; THL/6261/14.06.00/2021). Data access was not based on patient consent but was allowed for the legitimate purpose of a scientific study. Pseudonymized individual-level data were analyzed entirely within a secured environment provided by the Finnish Institute for Health and Welfare. To maintain confidentiality and in compliance with our data use agreement, we do not report exact numbers for population subgroups containing <5 people.

**Table 1:** Data sources by outcome measures.^a^

	Secondary Care Registry Hilmo	Primary Care Registry AvoHilmo	Prescription Center	Digital and Population Data Service Agency	University Hospital Electronic Records (data lakes)
CF diagnosis	X	X			X^b^
CF patient characterization	X	X		X	
Age at death				X	
Comorbidities	X	X			
Purchased medication			X		
Healthcare resource use	X	X	X		
*CFTR* mutation data					X
Reference cohort identification				X	
Reference cohort data	X	X	X		

CF: cystic fibrosis; CFTR: cystic fibrosis transmembrane conductance regulator.

a Permission for data handling was provided by the Finnish Social and Health Data Permit Authority Findata with diary numbers THL/6256/14.02.00/2020, THL/3657/14.06.00/2021, and THL/6261/14.06.00/2021.

b Diagnosis confirmation by clinicians.

Electronic data to identify the CF population are available from the beginning of 1969. The CF population included individuals who were alive on January 1, 2015, or born during the study period (January 1, 2015, to December 31, 2019) and who had ≥3 separate registry entries indicating a CF diagnosis based on *International Classification of Diseases* (*ICD*) codes (Hilmo: *ICD-8*273.0 for 1969–1986; *ICD-9*277.0 for 1987–1995; *ICD-10* E84 for 1996–2019; AvoHilmo: *ICD-10* E84 for 2011–2019), which were individually verified by expert clinicians. Epidemiology was described using the diagnosis date, which was defined as the date of the first record indicating CF.

After defining the CF cohort, 5 controls per each person with CF were identified by matching on the following criteria: 1) sex, 2) age at index date (±6 months), and 3) place of residence. Control individuals were required to have ≥1 record in the primary or secondary care registries between 1970 and 2019 and no diagnosis of CF per *ICD*. The index date for the 5 controls was defined as the diagnosis date for their matched CF counterpart. Each individual could act as a control for only 1 person with CF; individuals in the CF cohort could not act as controls. Disease burden in terms of comorbidities, medication use, and healthcare resource use among people with CF was compared to that in the reference population.

To describe disease burden, the start of the study period was defined as January 1, 2015 (for individuals diagnosed prior to the study period), or the date of CF diagnosis (for those diagnosed during the study period); the end of the study period was the date of death or December 31, 2019 (whichever occurred first). There were no outcome measures other than the end of the follow-up period. Drug purchases were captured based on anatomical therapeutic chemical (ATC) codes, and comorbidities were identified using *ICD-10* codes recorded during follow-up. Healthcare resource use was estimated as the number of visits (outpatient, inpatient, intensive care unit, and emergency department) divided by the follow-up time in years.

### 2.1 Statistical analysis

Analyses of individual-level, pseudonymized data were conducted with R (version: 4.1.3). The overall prevalence in 2019 and prevalence per 5 university hospital–specific catchment areas (Helsinki, Kuopio, Oulu, Tampere, and Turku) were calculated for the identified CF cohort using the following formula:

$$p=\frac{n_{2019}}{population_{2019}}\cdot 100,000$$

The numerator (*n*_2019_) is the number of people with CF in the study cohort who were alive in 2019. The denominator (*population*_2019_) is the population of Finland in 2019, which was estimated to be 5,525,292 ^7^.

Characteristics such as sex, age in 2019, and age at diagnosis were reported for people with CF. Age in 2019 and at diagnosis were reported using summary statistics of age in years and the proportion of people with CF in the age groups of ≤18 years, ≥19 years, and, in some cases, 0 through 2 years, 3 through 10 years, and 11 through 18 years. Age at death was reported using descriptive summary statistics. Disease frequencies (number of different diagnoses by *ICD-10* codes) and drug use (drug purchases by ATC codes) were summarized as the proportion of individuals per category. The difference between the CF cohort and the reference population was evaluated by side-by-side box plot. In all healthcare resource use analyses, people with CF and their matched controls were analyzed using pairwise methods. Median intensities were used for matched controls to avoid the influence of outliers. If a person with CF or control did not have any visits during the follow-up period, the number of visits—and, therefore, healthcare resource use intensity—was defined as zero.

## 3 Results

In total, 102 people were verified to have CF, included in the CF cohort, and alive at the end of the study period. Prevalence per 100,000 inhabitants was 1.85 (N = 102) in 2019 and was highest in the Tampere University Hospital–specific catchment area (2.88; n = 26) and lowest in the Oulu University Hospital–specific catchment area (1.22; n = 9) (Table 2). The intended matching ratio was 1:5 (i.e., 510 controls for 102 people with CF), but in total, 508 age, sex, and residence-matched controls for the 102 people with CF were identified. People with CF did not differ from controls in terms of prespecified matching criteria. The mean age difference was 2.3 days (SD: 16.2 days; median: 0.6 days). Sex and place of residence were 100 % equivalent.

**Table 2:** Prevalence and characteristics in people with CF in 2019.

People with CF in Finland^a^	n	%	Prevalence (per 100,000)
	102	100.0	1.85
Region (university hospital–specific catchment areas)			
Helsinki	33	32.4	1.51
Kuopio	13	12.7	1.62
Oulu	9	8.8	1.22
Tampere	26	25.5	2.88
Turku	21	20.6	2.42
Sex			
Male	56	54.9	
Female	46	45.1	
Age at end of 2019, years			
Mean (SD)	24.8 (16.1)		
Median (IQR)	21.5 (12.3–36)		
Age at diagnosis,			
0–2 years	61	59.8	
3–10 years	20	19.6	
11–18 years	6	5.9	
≥19 years	15	14.7	
Genotype			
*F*/*F*	24	23.5	
*F*/MF	23	22.5	
Unclassified/Other	55	53.9	

CF: cystic fibrosis; *F*/*F*: homozygous *F508del*; IQR: interquartile range; *F*/MF: heterozygous *F508del* with any minimal function mutation.

a People with CF were matched to 508 controls by region, sex and age; controls did not differ from people with CF in terms of prespecified matching criteria.

Approximately half (55 %) of identified people with CF were men. Median age of people with CF was 21.5 years (mean: 24.8 years; SD: 16.1 years). Median age at diagnosis was 1 year (interquartile range: 0–6 years). Diagnosis was before 2 years of age in 60 % and before 10 years of age in 80 % of people with CF Nearly half had an *F508del* mutation (Table 2).

Fewer than 5 individuals in the CF population died before the end of the study period. Median age at death was 31.4 years. Due to the small sample size and to protect individual privacy, no other descriptive values are reported for those who died. No age-matched controls died during the study period.

**Fig. 1:**
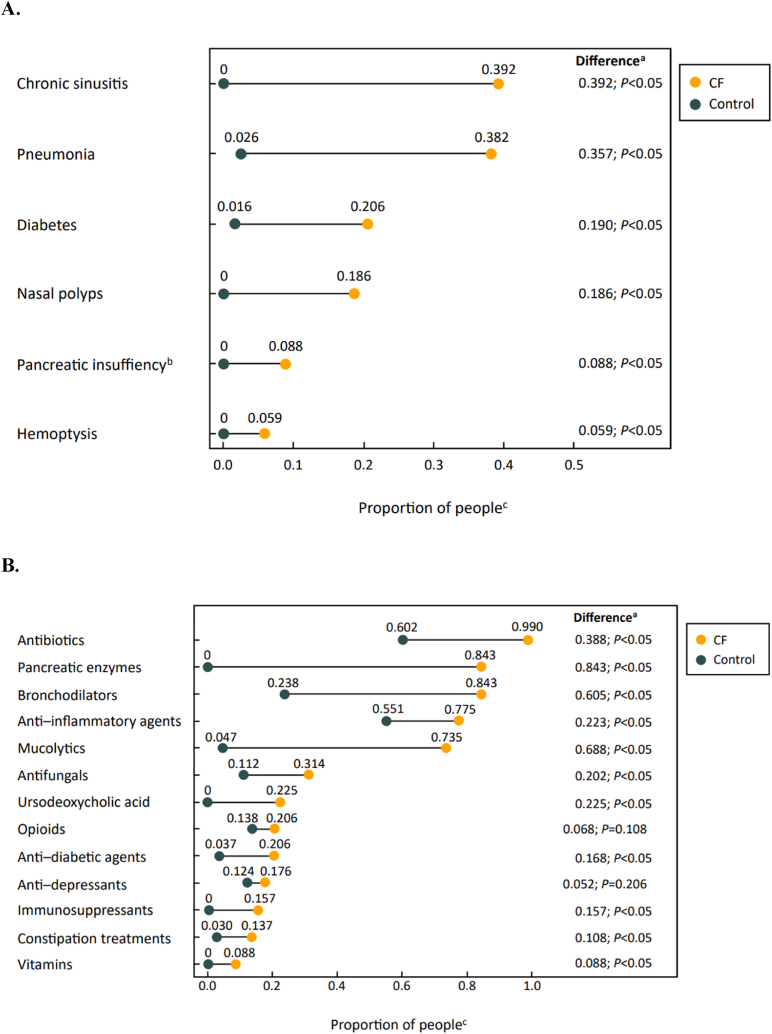
Most common comorbidities (A) and purchased medications (B) in the CF cohort (N = 102) and control population (N = 508) over the study period of 2015–2019. CF: cystic fibrosis; *ICD*: *International Classification of Diseases*. ^a^ The proportion test was used to test whether the difference between the CF and control populations was statistically significant. This test was implemented using R prop. test (R Foundation for Statistical Computing; 2018. Available from: https://www.R-project.org/). P values were not adjusted for multiple comparisons. ^b^ The proportion of people with comorbidities, including pancreatic insufficiency, was based on diagnoses (*ICD-10* codes) recorded for the person during the study period. The proportion of people who purchased pancreatic enzymes, which are used to treat pancreatic insufficiency, during the study period is reported in Fig. 1B ^c^ For the control population, the proportion of people with a comorbidity was calculated with the equation *n*_*yes*_/(*n*_*yes*_ + *n*_*no*_), where *n*_*yes*_ is the number of people with that comorbidity and *n*_*no*_ is the number of people without that comorbidity. The proportion of people in the control population with a purchased medication was calculated similarly.

Of the most common comorbidities recorded for people with CF during follow-up, chronic sinusitis (39.2 %), nasal polyps (18.6 %), pancreatic insufficiency (8.8 %), and hemoptysis (5.9 %) were only seen in people with CF (Fig. 1A). Pneumonia and diabetes (not restricted to CF-related diabetes) were more common in people with CF than controls (38.2 % and 2.6 %, respectively, for pneumonia; 20.6 % and 1.6 %, respectively, for diabetes).

The most commonly purchased medications among people with CF were antibiotics (99.0 %), pancreatic enzymes (84.3 %), bronchodilators (84.3 %), anti-inflammatory agents (77.5 %), and mucolytics (73.5 %). Among controls, the most commonly purchased medications were antibiotics (60.2 %) and anti-inflammatory agents (55.1 %) (Fig. 1B).

**Fig. 2:**
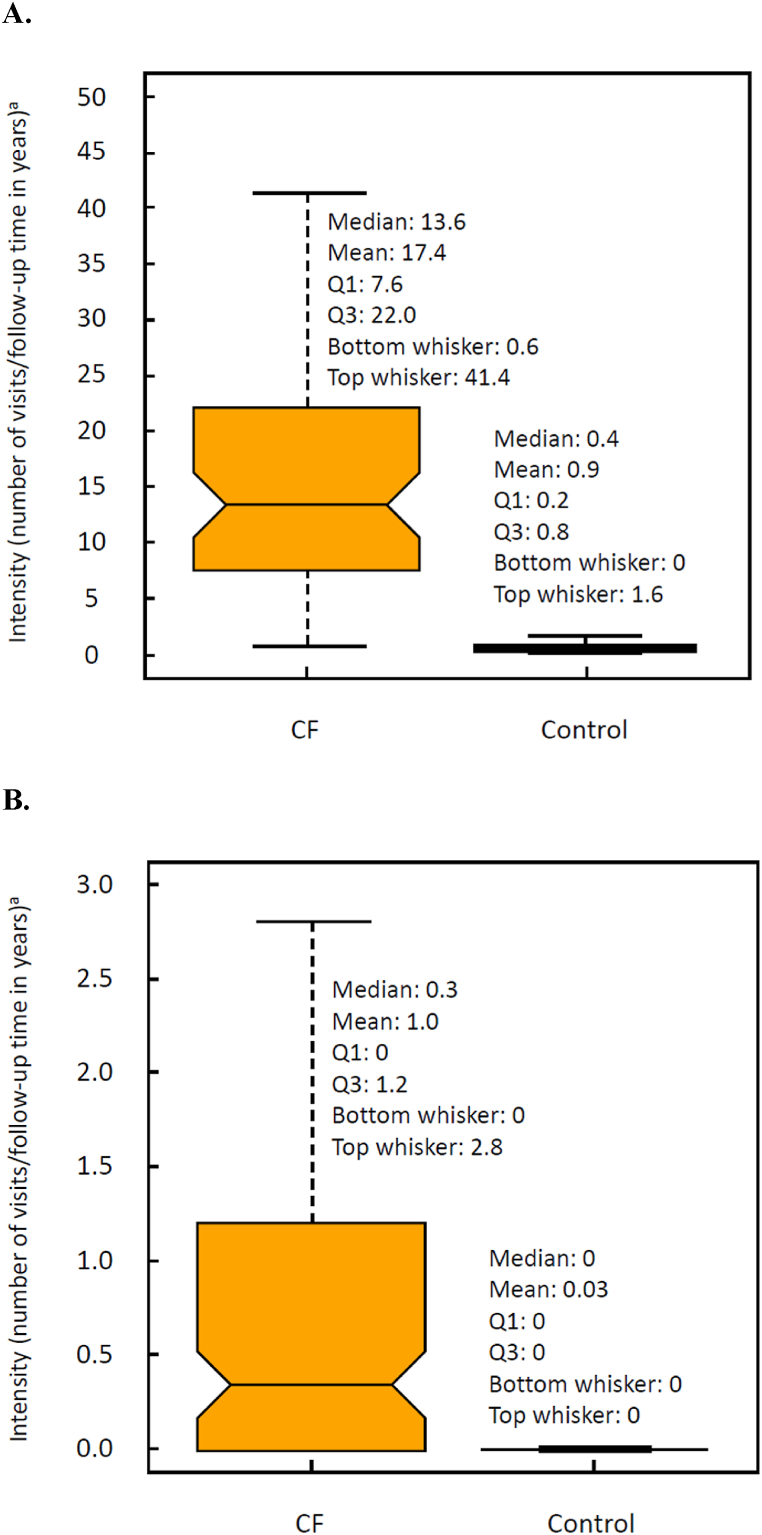
Outpatient (A) and inpatient (B) hospital visits for the CF cohort (N = 102) and control population (N = 508) over the study period of 2015–2019. CF: cystic fibrosis; IQR: interquartile range; Q1: quartile 1; Q3: quartile 3. ^a^ Data are presented using box plots, with the horizontal line inside the shaded area indicating the median and the upper and lower borders of the shaded area indicating Q3 and Q1, respectively. Whiskers above and below the shaded area indicate the maximum and minimum values, respectively, of the set without outliers. Top outliers are values larger than Q3 + 1.5 × IQR, and bottom outliers are values smaller than Q1 – 1.5 × IQR. Outliers are not shown in the box plots to preserve anonymity (i.e., a point would represent an individual).

In general, use of healthcare resources was notably higher among people with CF than among controls. The median number of outpatient hospital visits among people with CF and controls was 13.6 (mean: 17.4; SD: 14.5) and 0.4 (mean: 0.9; SD: 3.3), respectively (Fig. 2A). For inpatient hospital visits, the median number of visits among people with CF was 0.34 (mean: 1.0; SD: 1.66) vs 0 for controls (mean: 0.03; SD: 0.14) (Fig. 2B). However, there was no difference in the length of inpatient hospital stay between people with CF (median: 2 days; mean: 4.54 days; SD: 6.60 days) and controls (median: 2 days; mean: 4.53 days; SD: 6.87 days). The number of emergency department visits and intensive care unit visits was close to zero for both people with CF and controls.

## 4 Discussion

Here, we describe CF epidemiology and healthcare use among the CF population in Finland from 2015 through 2019, which is prior to the availability of CFTRm. In 2019, the prevalence of CF was 1.85 per 100,000 inhabitants, and the median age of people with CF was 21.5 years. During the study period, the median age at diagnosis was 1 year, and the median age at death was 31.4 years (n < 5). Comorbidities most common in the CF population were related to lung and pancreatic disease symptoms. Antibiotic use was notably high in the CF population, and the number of hospital visits was much greater than in the matched control population. This study provides a description of the demographic and clinical characteristics of the Finnish CF population and characterizes their healthcare utilization from 2015 through 2019, which is essential to understanding the burden of CF in Finland prior to the availability of CFTRm.

The prevalence of CF in Finland was notably lower than that reported in many other Western European countries in 2004 (e.g., per 100,000: Austria, 8.39; Belgium, 10.3; Ireland, 29.8; Sweden, 4.03) [15]. The relatively low prevalence may be due to the unique heritage of the Finnish population resulting from regional isolation and sparse inhabitation. This may also explain the observed regional variation, in which prevalence was notably higher in the Tampere University Hospital–specific catchment area (2.88) than in the Oulu University Hospital–specific catchment area (1.22). The relatively low prevalence may also be, in part, due to delayed diagnosis.

Unlike in many other countries, newborns in Finland are not systematically screened for CF. While 60 % of people with CF in this study were diagnosed by the age of 2 years, a substantial proportion (40 %) were not. Consistent with this finding, the median age at diagnosis was 1 year, which is substantially older than the median of 0.31 years reported in other European countries [16]. The implementation of newborn screening, including characterization of *CFTR* mutations, may be increasingly important in reducing the burden of CF in Finland.

Studies have shown that CFTRm are able to slow the progression of disease, including advanced forms, and may improve sweat chloride concentration (a marker of CFTR channel function) and protect and preserve pancreatic function when prescribed for young people with responsive CFTR mutations [2,[17], [18], [19]]. Our study indicates that approximately half of patients with CF in Finland have at least one copy of the *F508del* mutation and thus are eligible for CFTR modulator therapy. CFTRm treatment may not reverse organ damage that has already occurred; therefore, many people with CF will continue to require close monitoring and supportive treatment for ongoing CF complications. However, numerous clinical trials and long-term, real-world evidence studies have demonstrated that CFTRm, such as ivacaftor, can reduce the burden of CF by providing significant benefits in lung function, improved nutritional status, decreased healthcare use, improved quality of life, decreased mortality, and reduced lung transplants [20,21]. With the availability of highly effective treatment, early disease diagnosis and recognition of the mutation type have become even more crucial.

The median age at death (31.4 years) observed in the CF population in Finland during the follow-up period, prior to the availability of CFTR modulators, was much lower than that in the general population of Finland, which was 85 years in women and 77 years in men in 2019 [22]. The median age at death in people with CF in Finland in this study is not unlike that in other Western countries during a similar time period: 32.4 years in the US [23], 31 years in the UK [24], and 30.0 years across Europe [16]. The number of deaths among people with CF during the follow-up period in this study was small (<5), and the median was calculated only for those who died in this time period; therefore, this value may not provide a valid estimate of life expectancy in this population.

Comorbidities reported more often in the CF population vs the control population during the study period aligned with the disease pathophysiology of affecting the airways and respiratory system (chronic sinusitis, pneumonia, nasal polyps, and hemoptysis) and pancreas (diabetes and pancreatic insufficiency). The medications purchased most often were notably different between the CF population and the control population, and they were aligned with the standard of care for CF to address pulmonary symptoms (e.g., antibiotics, bronchodilators, mucolytics) and treat pancreatic insufficiency (pancreatic enzymes). Use of antibiotics, while common in controls (60.2 %), was notably high in the CF population, with 99 % of people with CF reporting use. Among people with CF, the high frequency of pancreatic enzyme replacement therapy in Finland is consistent with pancreatic enzyme use reported across Europe [16].

As expected, the frequency of outpatient and inpatient hospital use was notably greater in the CF population vs the control population, with nearly 20 times more outpatient and over 30 times more inpatient visits seen in the CF population. The majority of hospitalizations in CF are due to pulmonary exacerbations (PEx), generally characterized as an acute worsening of respiratory symptoms requiring intervention [25]. The mean number of hospitalizations observed in the Finnish CF population is higher than the number of exacerbations observed in the US population prior to availability of the first CFTRm, ivacaftor, in 2012 (0.65 exacerbations per year according to 2010 US registry data [26]), which may reflect differences in disease severity and/or management of PEx. Although people with CF had more hospital visits than the control population, the length of hospital stay was not different between these 2 groups (median duration: 2 days). The short length of stay and unexpected finding that it was similar in both groups may be partially due to the availability of outpatient treatment options in Finland, such as home hospitals, which provide healthcare for people in their homes [27]. People with CF in Finland may be familiar with the home hospital system and thus more likely to be discharged from hospitals so they can transition to home hospitals earlier than people without CF.

A strength of this study is the use of unique data linkages to overcome the lack of a specific CF registry in Finland. Data from several national registries were combined with data from five university hospitals using a unique personal identifier. This combined data source provides both clinical and health-care utilization data for a nearly complete population of people in Finland diagnosed with CF during the time period studied. Finland joined the European Cystic Fibrosis Society Patient Registry (ECFSPR) and began transferring data in early 2022 [28]. Joining ECFSPR was an important milestone in standardizing reporting of demographic and clinical data for Finnish people with CF, allowing analysis in this larger European context; however, the data linkages available through Findata provide information beyond that available in the ECFSPR and remain a rich source for future research.

This study also has several limitations. Real-world studies in CF are challenged by accurately identifying individuals with CF. In this study, administrative data were used to identify people with and without CF based on *ICD* codes. Diagnosis in the CF cohort was confirmed by expert clinicians based on results of genetic and sweat tests available in the hospital data. Misclassification of people with CF into the general population cohort cannot be ruled out; however, the proportion of misclassified people with CF should be very low. We also note that the matched control population allowed us to compare the disease burden in people with CF to that in a demographically similar non-CF population; however, the matched control cohort represented a limited sample of the Finnish population and cannot be assumed to be fully representative of the overall population. Moreover, because the assessment and recording of comorbidities were not undertaken for research purposes, some reporting may be incomplete, as seems to be the case with pancreatic insufficiency recorded using *ICD* codes.

This study was not intended to describe the complete burden of CF on individuals, their caregivers, or society in Finland. CF is a progressive disease that leads to declining health and premature death [4], with the associated societal burden related to disability and loss of productivity. CF also places a significant economic and quality-of-life burden on people with CF, their families, and society, due to various physical, psychiatric, and social consequences of this severe disease [1,4,29,30]. Exploration of these elements of CF burden is beyond the scope of the current analysis. However, an examination of societal and economic outcomes and the impact of CFTRm may be of interest for future studies.

Evidence of delayed CF diagnosis in the Finnish population indicates the need for a newborn CF screening program across Finland to ensure that people with CF are diagnosed and begin receiving treatment for this life-shortening, burdensome disease as early in life as possible. Higher numbers of comorbidities and greater resource and medication use in people with CF vs the control population suggest that early investment in efficacious disease management and improved access to appropriate treatment could lead to long-term savings, reduced disease burden, and, potentially, improved quality of life. At the end of the study period (December 2019), CFTRm were not yet in use in Finland. CFTRm have been available with reimbursement in Finland since spring 2021, and at the time of preparation of this manuscript, almost all eligible people with CF were receiving CFTRm. In future studies, it will be interesting to see how this new medication has affected the life expectancy and disease burden in people with CF in Finland.

## 5 Conclusions

First, by linking data from various national registries, we showed that use of medicines such as antibiotics is higher in the CF population than in the matched control population, and people with CF have notably more healthcare contact. Second, the prevalence of CF in Finland is remarkably lower than that in most other Western countries. Finally, the age at diagnosis is higher in Finland than in countries with newborn screening for CF but is similar to that in countries that do not screen newborns.

## Funding statement

This study was supported by Vertex Pharmaceuticals Incorporated. Sponsor-affiliated authors of this manuscript were involved in the study design, analyses, and interpretation of the results, with collaboration from the other authors.

## Disclosures are as follows

**KM** reports speaker fees from Vertex Pharmaceuticals. **MP**, **TP**, **PR**, **EH**, **AA**, and **TD** report no conflicts of interest. **VE** reports consulting and speaker fees from Vertex Pharmaceuticals. **KS** and **LJM** are employees of Vertex Pharmaceuticals and may own stock or stock options in that company.

## Ethics statement

Data access was not based on patient consent but was allowed for the legitimate purpose of a scientific study. Permission for data handling was provided by Findata (diary numbers: THL/6256/14.02.00/2020; THL/3657/14.06.00/2021; THL/6261/14.06.00/2021). Pseudonymized individual-level data were analyzed entirely within a secured environment provided by the Finnish Institute for Health and Welfare. Informed consent was not required for this study because patient data were analyzed in an anonymous format, and only summary statistics were presented. We do not report exact numbers for population subgroups containing <5 people, so that individuals cannot be recognized.

## Data sharing statement

The research data are confidential.

## CRediT authorship contribution statement

**Kirsi Malmivaara:** Writing – review & editing, Writing – original draft, Methodology, Formal analysis, Data curation, Conceptualization. **Mari Pölkki:** Writing – review & editing, Writing – original draft, Formal analysis. **Tuire Prami:** Writing – review & editing, Writing – original draft, Formal analysis. **Paavo Raittinen:** Writing – review & editing, Writing – original draft, Formal analysis, Data curation. **Eija Heikkilä:** Writing – review & editing, Writing – original draft, Methodology, Data curation, Conceptualization. **Antti Aalto:** Writing – review & editing, Writing – original draft, Formal analysis. **Teija Dunder:** Writing – review & editing, Writing – original draft, Formal analysis, Data curation. **Varpu Elenius:** Writing – review & editing, Writing – original draft, Formal analysis, Data curation. **Kristina Sandström:** Writing – review & editing, Writing – original draft, Formal analysis. **Lisa J. McGarry:** Writing – review & editing, Writing – original draft, Methodology, Formal analysis, Conceptualization.

## Declaration of competing interest

The authors declare the following financial interests/personal relationships which may be considered as potential competing interests:Kirsi Malmivaaraa reports administrative support, article publishing charges, and writing assistance were provided by Vertex Pharmaceuticals Incorporated. Mari Polkki reports administrative support, article publishing charges, and writing assistance were provided by Vertex Pharmaceuticals Incorporated. Tuire Prami reports administrative support, article publishing charges, and writing assistance were provided by Vertex Pharmaceuticals Incorporated. Paavo Raittinen reports administrative support, article publishing charges, and writing assistance were provided by Vertex Pharmaceuticals Incorporated. Eija Heikkila reports administrative support, article publishing charges, and writing assistance were provided by Vertex Pharmaceuticals Incorporated. Antti Aalto reports administrative support, article publishing charges, and statistical analysis were provided by Vertex Pharmaceuticals Incorporated. Teija Dunder reports administrative support, article publishing charges, and writing assistance were provided by Vertex Pharmaceuticals Incorporated. Varpu Elenius reports administrative support, article publishing charges, and writing assistance were provided by Vertex Pharmaceuticals Incorporated. Kristina Sandstrom reports administrative support, article publishing charges, and writing assistance were provided by Vertex Pharmaceuticals Incorporated. Lisa J. McGarry reports administrative support, article publishing charges, and writing assistance were provided by Vertex Pharmaceuticals Incorporated. Kirsi Malmivaaraa reports a relationship with Vertex Pharmaceuticals Incorporated that includes: speaking and lecture fees. Varpu Elenius reports a relationship with Vertex Pharmaceuticals Incorporated that includes: consulting or advisory and speaking and lecture fees. Kristina Sandstrom reports a relationship with Vertex Pharmaceuticals Incorporated that includes: employment and equity or stocks. Lisa McGarry reports a relationship with Vertex Pharmaceuticals Incorporated that includes: employment and equity or stocks. If there are other authors, they declare that they have no known competing financial interests or personal relationships that could have appeared to influence the work reported in this paper.
